# Retinoic acid reduces human neuroblastoma cell migration and invasiveness: effects on DCX, LIS1, neurofilaments-68 and vimentin expression

**DOI:** 10.1186/1471-2407-8-30

**Published:** 2008-01-29

**Authors:** Elio Messi, Maria C Florian, Claudio Caccia, Mariarosa Zanisi, Roberto Maggi

**Affiliations:** 1 Institute of Endocrinology, Centre of Oncological Endocrinology, University of Milan, Via Balzaretti 9, 20133 Milan, Italy; 2 Unit of Genetics of Neurodegenerative and Metabolic Diseases, Fondazione IRCCS Istituto Neurologico Carlo Besta, Via Celoria 11, 20133 Milan, Italy

## Abstract

**Background:**

Neuroblastoma is a severe pediatric tumor, histologically characterised by a variety of cellular phenotypes. One of the pharmacological approaches to neuroblastoma is the treatment with retinoic acid. The mechanism of action of retinoic acid is still unclear, and the development of resistance to this differentiating agent is a great therapy problem.

Doublecortin, a microtubule-associated protein involved in neuronal migration, has recently been proposed as a molecular marker for the detection of minimal residual disease in human neuroblastoma. Nevertheless, no information is available on the expression of doublecortin in the different cell-types composing human neuroblastoma, its correlation with neuroblastoma cell motility and invasiveness, and the possible modulations exerted by retinoic acid treatment.

**Methods:**

We analysed by immunofluorescence and by Western blot analysis the presence of doublecortin, lissencephaly-1 (another protein involved in neuronal migration) and of two intermediate filaments proteins, vimentin and neurofilament-68, in SK-N-SH human neuroblastoma cell line both in control conditions and under retinoic acid treatment. Migration and cell invasiveness studies were performed by wound scratch test and a modified microchemotaxis assay, respectively.

**Results:**

Doublecortin is expressed in two cell subtypes considered to be the more aggressive and that show high migration capability and invasiveness.

Vimentin expression is excluded by these cells, while lissencephaly-1 and neurofilaments-68 are immunodetected in all the cell subtypes of the SK-N-SH cell line. Treatment with retinoic acid reduces cell migration and invasiveness, down regulates doublecortin and lissencephaly-1 expression and up regulates neurofilament-68 expression. However, some cells that escape from retinoic acid action maintain migration capability and invasiveness and express doublecortin.

**Conclusion:**

a) Doublecortin is expressed in human neuroblastoma cells that show high motility and invasiveness;

b) Retinoic acid treatment reduces migration and invasiveness of the more aggressive cell components of SK-N-SH cells;

c) The cells that after retinoic acid exposure show migration and invasive capability may be identified on the basis of doublecortin expression.

## Background

Neuroblastoma (NB), the most common extracranial solid paediatric tumor, is responsible for approximately 15% of cancer deaths occurring in childhood. In older patients the prognosis is still poor although the clinical course may be protracted [1]. Consistent with their origin from neural crest-derived multipotent precursors, NBs are often composed of multiple cellular phenotypes. This heterogeneity is retained in some cell lines derived from these tumors.

In SK-N-SH cell line, derived from a bone marrow metastasis, at least three cell types are present: the sympathoadrenal neuroblasts (N), the substrate-adherent non-neuronal (S), and the intermediate (I) cells. [2,3]. The N-type are small rounded cells with short neuritic processes, whereas the S-type are large, flat and strongly attached to the substrate. The I-type have a morphology intermediate between that of N and S, are moderately adherent to the substrate and show small cell body with or without neuritic elongations. In addition, the I-type cells have the ability of forming aggregates in culture [3]. The three cell types differ also in the capability to induce the tumor; it has been reported that N-type cells are malignant while the S-type are not, but surprisingly, the I-type cells have the greatest malignancy [2]. Moreover, it has been hypothesized that the I-type, may be recognized as tumor stem cells, capable of both self-renewal and bidirectional differentiation, being the progenitor of both N- and S-type [4].

The current treatment of NB may involve surgery, chemotherapy, radiation therapy, megatherapy with stem cell rescue and biological approaches [5]. Arrest of cell growth and morphological differentiation can be observed after retinoic acid (RA) treatment at particular stages of progression of the disease, but the precise mechanism of action of RA remains uncharted; moreover, resistance to RA represents a significant drawback to its clinical utility.

In recent years, a number of genetic and biological features have been investigated in the effort to identify tumor markers that would improve cure rates.

Doublecortin (DCX), a microtubule associated protein, has been proposed as a new molecular marker to detect minimal residual disease in human NB, since it has been detected in all the tumors analyzed and it appears to be more efficient than tyrosine hydroxylase [6].

Moreover, DCX, semaphorin3B and SPARC (Osteonectin), three genes that play key roles in cellular migration processes, have been indicated as prognosticators of poor prognosis in human glioblastoma [7].

Recently, it has also been reported that DCX is present in tumors of the human central and peripheral nervous system [8] and that is preferentially expressed in invasive human brain tumors [9].

DCX is the product of the X-linked gene *doublecortin *(Xq22.3-Xq23) and is required for neuroblastic migration during development of the cerebral cortex [10]. Mutations in this gene result in a deficient neuronal migration that gives rise to the X-linked lissencephaly in males or to the subcortical laminar heterotopia (doublecortex) in female patients [11,12]. DCX is expressed in migrating neurons throughout the central and peripheral nervous system during embryonic and postnatal development, regulates the microtubule cytoskeleton and it is involved in dynamic morphological changes [13-15] and in nuclear translocation occurring during neural cell migration. In this process it acts synergistically with lissencephaly-1 (LIS1), another microtubule associated protein codified by the gene *Lis1*, located on chromosome 17p.13.3 [16,17]. LIS1 localizes predominantly to the centrosome and acts with DCX for the correct nucleus-centrosome coupling required for normal neuronal migration [17].

To date, the presence of DCX in human NB has been evaluated only by real-time RT-PCR in tumor samples [6], and by Western blot (WB) analysis in SH-SY5Y cell line [18]. However it is not known whether DCX is expressed in all the different cell-types (S, N and I) composing the tumor or it is a characteristic of some of them, and whether its interacting protein LIS1 is also expressed in the human NB.

In the present study we have analysed by immunofluorescence (IF) the presence of DCX and LIS1 in the different cell subtypes that constitute the SK-N-SH neuroblastoma cell line. We have also correlated the intracellular localization of these two proteins with tubulin and with two intermediate filaments: vimentin (VIM) and neurofilament-68 (NF-68) that have been utilized as possible cell-subtype specific markers. Furthermore, we have investigated by IF and WB analysis whether a treatment with the differentiating agent RA might induce modifications on DCX, LIS1, VIM and NF-68 expression. In addition, motility and invasiveness of SK-N-SH cells have been studied by wound scratch test [19] and by a modified microchemotaxis assay [20], so as to evaluate the capability of RA to influence these parameters.

## Methods

### Cell cultures

Human SK-N-SH cells were grown as a monolayer in Eagle's Minimum Essential Medium (MEM) supplemented with 10% of heat-inactivated Foetal Bovine Serum (FBS from Gibco, Invitrogen), 1% non essential amino acids (NEAA), 2 mM L-glutamine, 1 mM sodium pyruvate, penicillin (20 units/ml) and streptomycin (20 mg/ml), and were maintained at 37°C in a saturated humid atmosphere with 5% CO_2_. As they approached confluence, the cells were split following treatment with Trypsin-EDTA. The seeding density varied according to the type of experiment.

### DCX sequence analysis

Genomic DNA from the SK-N-SH cell line was prepared using standard procedures [21]. Sequence analysis of the *DCX *gene coding regions was performed essentially as described previously [11]. Briefly, the 7 *DCX *coding exons (exons 1C-7; NCBI Ac. Nos. AJ005592-AJ005597) and their flanking intron sequences were amplified by PCR and directly sequenced using an automated sequencing apparatus (3100 Genetic Analyzer, Applied Biosystems, Foster City, CA).

### Treatment with Retinoic Acid

All-*trans *RA (Sigma) was added to a final concentration of 10 μM, according to known pharmacological dosages used in phase I trials of RA administered orally to NB patients [22]. Moreover, it has been well established that addition of 10 μM RA to proliferating NB cells induces their differentiation [23]. To study RA effects on SK-N-SH cell line, cells were plated at a density of 5 × 10^5^cells/10 cm diameter Petri dish for WB at a density of 5 × 10^3^cells/13 mm diameter well for IF or alternatively at a density of 2 × 10^4^cells/13 mm diameter well for the scratch wound assay, and grown in MEM medium supplemented as described above. RA was first dissolved in absolute ethanol and then supplemented with DMSO to achieve a concentration of 10^-3 ^M in ethanol-DMSO (87.5%–12.5%). This stock solution was kept at -20°C. For each experiment RA was diluted at the desired concentration directly into the growth medium. Cells cultured in medium containing 0.1% of ethanol-DMSO (87.5%–12.5%) were used as control. The 10 μM RA treatment started on the second day after plating, concomitantly with the medium replacement. Cells were fed every 48 hr with control, or RA- containing fresh medium and the treatment was stopped 2, 4, 6 or 12 days after the first addition of RA. In order to investigate whether the effect of RA on DCX protein expression was reversible, we treated SK-N-SH cells with RA for 6 days; afterwards the cells were grown in control medium for 6 additional days.

### Antibodies

For both IF and WB the primary antibodies used were: anti-DCX (C-18 sc-8066; 1:500) and anti-LIS1 (H-300 sc-15319; 1:200) from Santa Cruz Biotechnologies, anti-vimentin (1:10,000) and anti-neurofilaments-68 (1:1,000) from Chemicon International and anti-α-tubulin (1:500) from Sigma.

### Immunofluorescence analysis

Cells were grown on 13 mm diameter coverslips, fixed in 4% paraformaldehyde (PFA), permeabilized in 0.2% Triton X-100 and then, the unspecific sites were blocked in 10% normal donkey serum (Sigma) in PBS. Subsequently, cells were incubated with the primary antibodies for 1 hr at room temperature and then rinsed with PBS. For triple-label IF, Cy3-conjugated anti-goat (for anti-DCX antibody), Cy2-conjugated anti-rabbit (for anti-LIS1, or alternatively for anti-neurofilaments-68 antibody) or AMCA-conjugated anti-mouse (for anti-α-tubulin or alternatively anti-vimentin antibody) secondary antibodies (Jackson ImmunoResearch) were used (1 hr at room temperature). The manufacturer guarantees the use of these secondary antibodies for multiple labelling procedures, since they are purified against cross-reactivity to other species. Nuclei were stained with DAPI (Sigma-Aldrich). Finally, the cells were washed in PBS before mounting using Mowiol 40–88 (Sigma-Aldrich).

### Western blot analysis

Cells were lysed with modified RIPA buffer (TRIS-HCl 50 mM pH7.4, NaCl 150 mM, Tryton X-100 0.1%, Na-deoxycholate, EDTA 0.1 M, SDS 1%) containing protease (ABESF, leupeptin and pepstatin) and phosphatase inhibitors (NaF 1 mM, Na_3_VO_4 _1 mM). For alkaline phosphatase treatment, cells were lysed with the aforementioned RIPA cell lysis buffer without phosphatase inhibitors (1 mM NaF and 1 mM Na_3_VO_4_) and then the cell lysates were incubated at 37°C for 30 minutes with calf intestinal alkaline phosphatase (CIP, 1 unit/μg of protein; Amersham Biosciences).

The total protein content for each sample was measured using the Bradford's method. 50–70 μg of proteins were separated on SDS-12% PAGE under reducing conditions and transferred onto nitrocellulose membranes. After blocking for 45 minutes at room temperature in 5% non-fat-milk in PBS, membranes were probed overnight at 4°C, with the primary antibodies diluted in 5% milk-PBS; they were subsequently washed with PBS and incubated with secondary antibodies coupled to horseradish peroxidase. The secondary antibodies used were HRP-conjugated- anti-goat (for anti-DCX antibody), anti-rabbit (for anti-LIS1 or for anti-neurofilamente-68 antibody), and anti-mouse (for anti-α-tubulin or anti-vimentin antibody) from Santa Cruz Biotechnologies. Detection and visualization of protein-bound antibodies was achieved using an enhanced chemiluminescence WB detection kit (ECL Plus, Amersham Biosciences). To quantify the intensity of the bands, membranes were scanned and analysed with the NIH Image software. All data are normalized against tubulin and expressed as percentage of the control.

### Wound Scratch Assay

In order to investigate SK-N-SH cell migration capability after RA treatment, a modified wound scratch assay was used [19,24]. Briefly, the treated and untreated cells were grown to confluent monolayer on 13 mm diameter glass coverslips. On the 6^th ^day of treatment, approaching 100% cell confluence, the monolayers were wounded by scratching the surface as uniformly as possible with a pipette tip. This initial wounding and the movement of the cells in the scratched area was photographically monitored using the Axiovert Zeiss 200 microscope with a 10× (NA 0.25) objective linked to a Coolsnap Es CCD camera (Roper Scientific – Crisel Instr. Rome) for 24 hr. This time interval has been chosen because it is shorter than SK-N-SH doubling time. Four different fields from each sample were considered for quantitative estimation of the distance between the borderlines and in each image four different equidistant points were measured in order to better estimate the real width of the wounded area. The migration rate is expressed as percentage of the control, and it was calculated as the proportion of the mean distance between both borderlines caused by scratching, to the distance which remained cell-free after re-growing [24]. Two independent series of experiments were performed in quadruplicates. To evaluate the correlation between DCX protein expression and cell migration capability, at the end of the photographic monitoring, the cells were fixed in 4% PFA and immunostained with anti-DCX antibody.

### Cell invasion assay

Cell invasion was determined by a modified microchemotaxis assay [20] using cell culture 24-well inserts with the bottom sealed by a 8 μm pore polycarbonate-filter coated with Matrigel (Chemicon International, Inc.). Briefly, 2.5 × 10^5 ^cells in 250 μl of serum-free MEM were seeded into the inserts. The lower chambers were filled with MEM additioned of 10%FBS. Chambers were incubated at 37°C in a 5% CO_2 _atmosphere for 48 hr. The cells that did not migrate through the filter were scraped from the insides of the inserts. The cells migrated to the underside of the coated filter were fixed in 4% paraformaldehyde for 5 min, washed two times with PBS and then processed for DCX IF analysis. Nuclei were stained with DAPI (Sigma). Alternatively, the migrated cells were incubated with cell detachment buffer and dissociated from the filter. The cells were then lysed and stained by CyQuant GR dye. Fluorescence was counted using VICTOR^3^™ multilabel readers (PerkinElmer^®^). Data are expressed as means ± standard error, N = 4.

### Image acquisition

Images were acquired using the UIC-Metavue 6.2.2 (UIC-Crisel Instr. Rome) imaging system on an Axiovert Zeiss 200 microscope. An oil immersion 63× (NA 1.4) or a 40× (NA 0.8) objective was used for IF images. To estimate the number of DCX-positive DCX^+^) or VIM-positive (VIM^+^) cells, at least 4 different images related to 3 control and 3 treated samples were considered. In each image acquired, the DCX^+ ^and the VIM^+ ^cells were counted. Data are graphically expressed as the ratio between the number of DCX^+ ^or VIM^+ ^cells to the number of the cells counted in total. For the wound scratch test analysis, images were acquired using a 10× (NA 0.25) objective. The distance between the borderlines of the scratched area was measured using the Region Measurement Function included in UIC-Metavue 6.2.2 (UIC-Crisel Instr. Rome) software.

### Statistical analysis

Results of quantitative analysis are presented as means ± standard error (S.E.); the number of samples varied between a minimum of 4 and a maximum of 8 depending on the experimental protocol. Control and treated samples have been compared by the one-sided unpaired Student's t-test or by ANOVA and using the GraphPad Prism software (version 4.0). The results are considered statistically significant versus the untreated cells, with a probability level of p < 0.05 (*), or statistically highly significant with a probability level of p < 0.01 (**).

## Results

### DCX is present in the N- and some I-type cells, but not in the S-type, of SK-N-SH cell line

**Figure 1:**
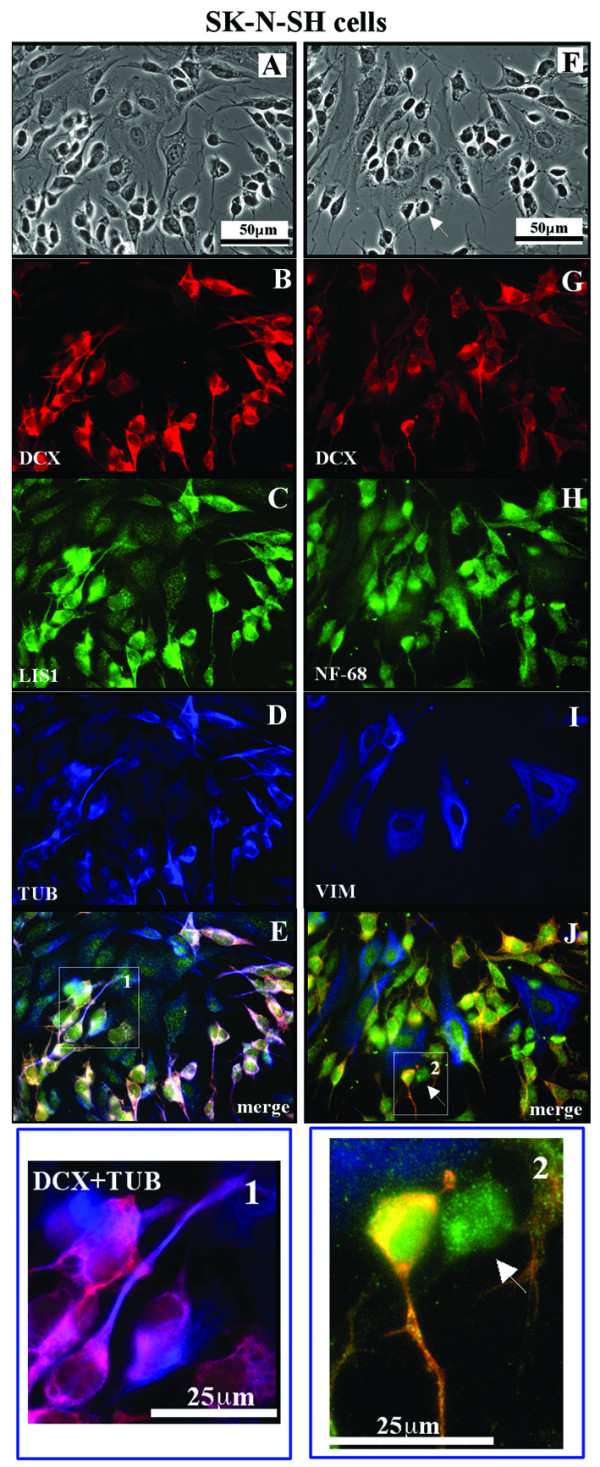
**DCX and vimentin are expressed by different SK-N-SH neuroblastoma cell subtypes**. The sets of figures **A**-**E **and **F**-**J **are representative of a series of images taken from SK-N-SH cells cultured in control conditions. PC analysis (panels **A **and **F**) clearly shows the heterogeneity of the cell population. The triple IF for DCX (**B**), LIS1 (**C**) and TUB (**D**) shows an immunoreactive signal for DCX only in the N-type and in some I-type cells. The anti-LIS1 antibody stains all the cell types. When co-expressed by the same cell, TUB and DCX colocalize both in the cell body and along the neuritic processes (see inset 1 of panel **E**). The triple IF for DCX (**G**), NF-68 (**H**) and VIM (**I**) shows that none of the DCX^+ ^cells is immunoreactive for VIM. Vimentin is present merely in the S-type and in some I-type DCX^- ^cells. Some other I-type cells do express neither DCX nor VIM (arrow in the inset 2 of panel **J**). The NF-68 protein is expressed by all the cell subtypes.

As already reported [2,3] the three different cell types composing SK-N-SH cell line were distinguishable by phase contrast (PC) microscopy analysis (Fig. 1A and 1F).

IF analysis for DCX shows a clear signal (DCX^+^) in all the cells with the N morphological phenotype and in some I-type, while it is absent (DCX^-^) in the S-type cells (Fig. 1). In the cells that express DCX this protein is present in the cell body and all along the neuritic elongations, and, as expected due to its microtubule associated protein nature, it colocalizes with tubulin (Fig. 1).

Since tumoral cells in culture may undergo genetic instability the possible presence of DCX deleterious mutations was considered and ruled out by direct sequence analysis (see Materials and Methods) of the SK-N-SH *DCX *gene coding regions (data not shown).

Since DCX can form etherodimers with LIS1, we also evaluated LIS1 expression and we found its immunoreactivity in all the SK-N-SH cell subtypes, with a stronger signal in DCX^+ ^cells (Fig. 1).

To further characterize the cell subtypes not only on the basis of DCX expression, we considered other markers, such as VIM and NF68.

As expected, S-type cells are immunopositive for VIM (VIM^+^) that is totally absent (VIM^-^) in the N-type cells (Fig. 1). A double staining for DCX and VIM clearly shows an exclusive expression in the different cell types; in fact, these proteins never colocalize in any cell type observed.

Among the cells with an intermediate morphology we identified at least three subpopulations: the first one shows immunoreactivity for DCX but not for VIM; the second one displays an opposite expression pattern of the two proteins; and the third one expresses neither DCX nor VIM (Fig. 1). NF-68 immunoreactivity is present in all the cells, even if the N-type shows a higher signal.

### Effects of exposure of SK-N-SH cells to retinoic acid

**Figure 2:**
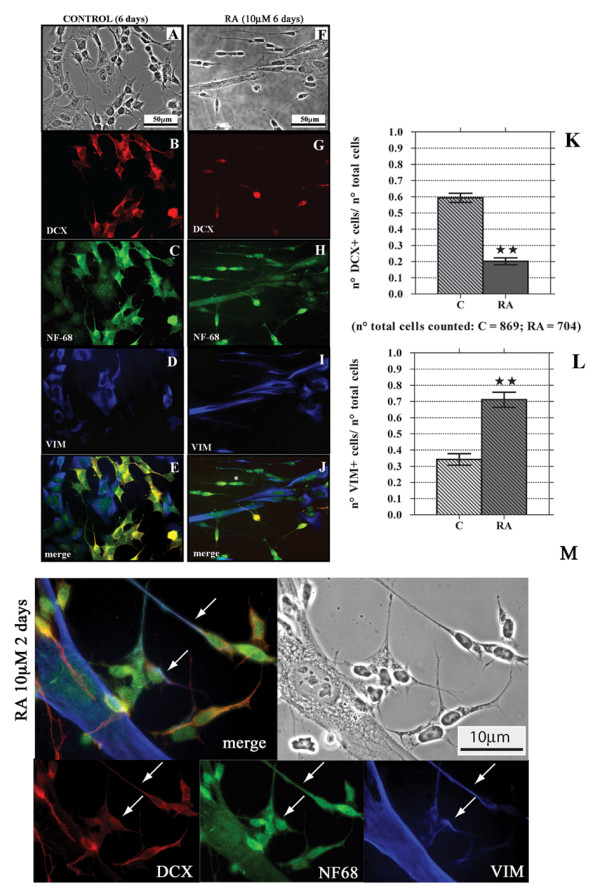
**Six days RA treatment reduces the number of DCX^+ ^cells and increases that of VIM^+ ^cells**. Figures here reported are representative of a series of images taken from SK-N-SH cells cultured in control conditions (**A**-**E**) or after six days RA treatment (**F**-**J**). PC analysis (panels **A **and **F**) shows morphological changes induced by RA. After 6 days RA treatment the DCX^+ ^cells are few (**G**) in comparison to the control (**B**), and they have the morphology of undifferentiated I-type cells. On the contrary is increased the number of VIM^+ ^cells and the differentiated N-type VIM^+ ^cells do not show immunoreactivity for DCX (**I**). Bar graphs show the decrease in the number of DCX^+ ^cells **(K) **and the increase in the number of VIM^+ ^cells **(L) **after RA treatment. Bars represent mean ± standard error, N = 8, double star = p < 0.01. Neither in control nor in 6 days RA treated samples there are DCX^-^/VIM^- ^cells immunopositive for both DCX and VIM, but as previously observed in control conditions (see fig.1), some DCX^-^/VIM^- ^cells are present after RA treatment (see the cell marked with *, panel **J**). As in the controls (**C**), NF-68 localizes in all RA treated SK-N-SH cells (**H**). It is important to note that after the first 2 days of treatment it is possible to observe N-type cells (white arrows) that start to express VIM while DCX is disappearing **(M)**.

As expected, 10 μM RA treatment induces a clear morphological differentiation of SK-N-SH N-type cells shown by the extension of their neuritic processes, Some S-type cells appear more elongate, but do not acquire a completely different phenotype (Fig 2).

In terms of DCX, after 2 (Fig. 2 panel M) or 4 days (data not shown) of RA exposure it is possible to observe a decreased stain in N-type cells while, in the same cell, the signal for VIM increases. After a longer (6 days) RA treatment, DCX immunoreactivity disappears from almost all of the N-type cells and it is replaced by VIM signal (Fig 2). The S-type cells remain DCX^-^, whereas VIM is always present even if the fluorescence signal is unchanged or slightly reduced (Fig. 2). A quantitative evaluation of the number DCX^+ ^and VIM^+ ^cells, regardless of their morphology, confirms that RA exposure leads to a decrease in the number of DCX^+ ^cells and to an increase in that of VIM^+ ^(Fig 2 panel K and L).

Regarding NF-68, RA treated cells show a more intense signal, in particular the N-type cells that underwent differentiation (Fig. 2).

After RA treatment, we also observe the same three subpopulations of I-type cells recognized in untreated cultures; moreover at this time interval, LIS1 immunostaining is apparently reduced, nevertheless it is still present in all cell types (data not shown).

**Figure 3:**
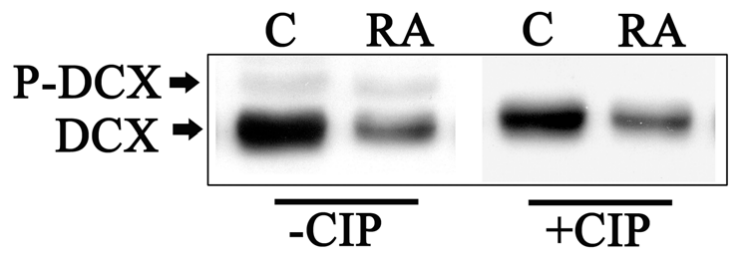
**DCX and phospho-DCX (P-DCX) expression in SK-N-SH cell line**. Lysates (50 μg/lane) of SK-N-SH control and 6 days RA treated cells were processed with or without calf intestine alkaline phosphatase (CIP) and analyzed by WB. DCX is a phosphoprotein and appears as a doublet that resolves to a single band after CIP treatment (+CIP) in both C and RA samples, indicating that the slower-migrating species is phosphorylated DCX (P-DCX).

WB analysis clearly shows double immunoreactive bands for DCX in SK-N-SH (Fig. 3). This doublet resolves to a single band after calf intestine alkaline phosphatase (CIP) treatment, indicating that the slower-migrating species correspond to phosphorylated DCX (Fig. 3), as already described [14,25]. However, since we observed preliminarily that RA treatment reduced the amount of both DCX forms, and in order to better quantify the decrease of its expression, in the following experiments we run the samples to get a single immunoreactive band for DCX, and utilized an anti-DCX antibody which reacts with both the phosphorylated and the unphosphorylated DCX forms [25].

**Figure 4:**
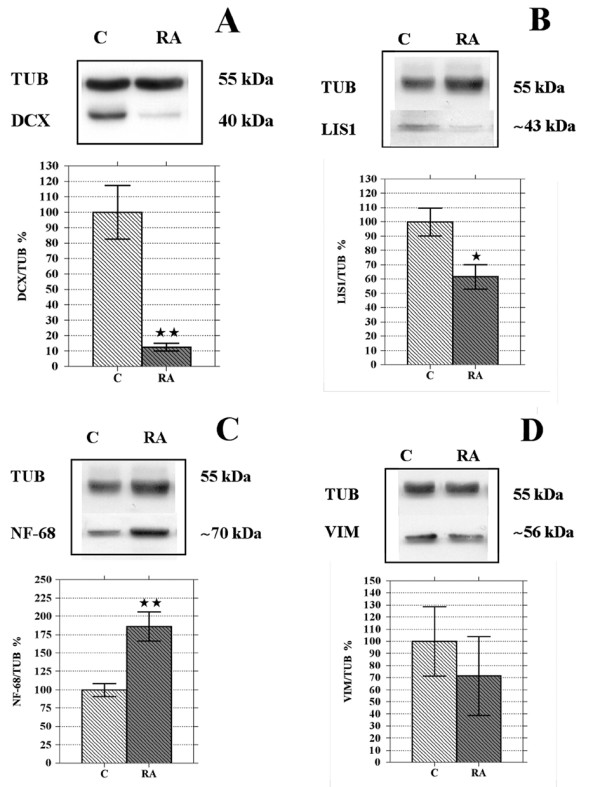
**Six days RA treatment lowers DCX and LIS1 protein levels while NF-68 content increases**. WB representative images and results of the densitometric analysis (**A**-**D**). After 6 days RA treatment the protein level of DCX (**A**) decreases by 87.4% and that of LIS1 (**B**) by 38.4%; NF-68 protein amount increases by 86.3% (**C**). Data related to VIM (**D**) show high variability and do not indicate any change in protein level after RA treatment. C, controls; RA, samples treated with 10 μM RA for 6 days. Bars represent mean ± standard error, N = 8 and data are expressed as a percentage of the control (100%); single star = p < 0.05; double star = p < 0.01.

Protein analysis reveals that the exposure of SK-N-SH cells to RA for 6 days significantly decreases both DCX (-87.4%) and LIS1 (-38.4%) levels, whereas NF-68 protein content (+86.3%) increases and VIM remains unchanged (Fig. 4).

**Figure 5:**
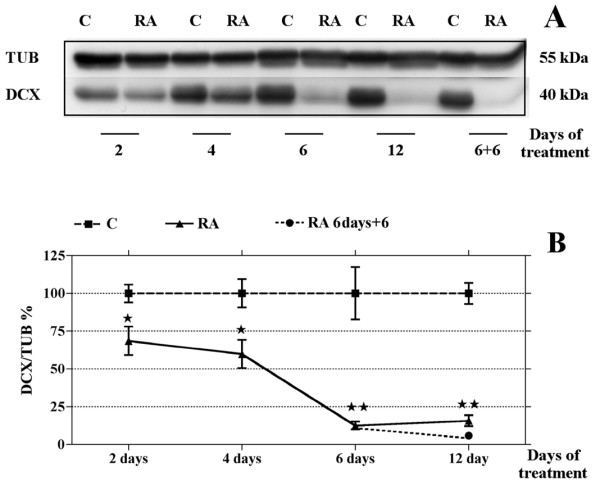
**DCX down regulation is time dependent and irreversible**. WB representative image **(A)**: lysates (50 μg/lane) of SK-N-SH control and RA treated cells at different time intervals (2, 4, 6, 6 plus 6 of withdrawal and 12 days) were analyzed by immunoblotting using an anti-DCX specific antibody; membranes were reprobed with anti-tubulin as a loading control. Quantification of DCX protein amount by scanning densitometry **(B)**: data are means ± standard error, N = 8, and are expressed as a percentage of the control (100%). After 2 or 4 days of RA treatment DCX levels are significantly decreased, but to a lesser extent than after a 6 days exposure to RA. Lengthening SK-N-SH exposure to RA up to 12 days does not modify the magnitude of the DCX reduction observed after 6 days of treatment. Moreover, in cells treated with RA for 6 days and then grown for 6 additional days in medium without RA (6 days+6), DCX protein amount remains at low levels; single star = p < 0.05; double star = p < 0.01; C, control, RA, samples treated with RA 10 μM.

A time-course WB analysis shows that 2- or 4 days treatment with RA significantly reduces DCX levels (Fig. 5) without modifying those of LIS1, VIM and NF-68 (data not shown). The DCX low level, observed after 6 days treatment, is not further diminished by extending RA exposure up to 12 days. Moreover, the down regulation exerted by 6 days RA exposure on DCX persists after an additional 6 days period following treatment withdrawal (Fig 5).

### Only DCX expressing cells show spontaneous motility after RA treatment

To evaluate the motility of SK-N-SH cells we have then analyzed their response to wound healing assay, either in control conditions or after RA treatment.

**Figure 6:**
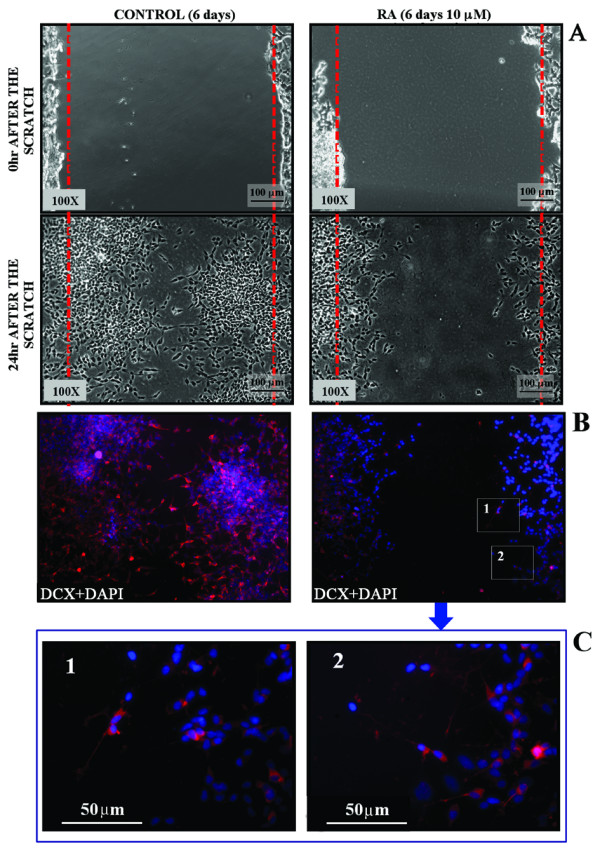
**RA treatment reduces SK-N-SK neuroblastoma cell motility**. (**A**) Representative PC images of untreated (Control) or 6 days RA treated cells which motility has been analyzed by scratch test. Images show the starting (0 hr after scratch) and the end (24 hr after scratch) point of the analysis. In control the distance between the borderlines becomes significantly shorter 24 hr after wounding, while it is still elevated in RA treated samples. **(B) **IF DCX analysis performed both in control and in RA treated cells 24 hr after wounding. In control samples the majority of invading cells are DCX^+ ^(red). Even if the migration rate of the treated cells is reduced, few of them move in and invade the scratched area; all the cells still migrating after 6 days RA treatment are DCX^+ ^(inset **1 **and **2 **of panel **B **are magnified in panel **C**). Nuclei are counterstained with DAPI (blue).

As shown in Fig. 6, untreated SK-N-SH cells are able to invade the scratched area that is fully re-colonised 24 hr later. IF analysis shows that all the cells moving in the scratched area are DCX^+ ^(Fig. 6).

Six days RA treatment strongly reduces the migration rate of the SK-N-SH cells. In fact, 24 hr after the scratch very few cells are in the scratched area and the distance between the borders of the wound is not significantly different from that measured immediately after the scratch (Fig. 6 panel A). The few cells that move into the scratched area are DCX^+^, most of them have short or no neurites, and small and refractile cell bodies (Fig. 6 panel B and C).

**Figure 7:**
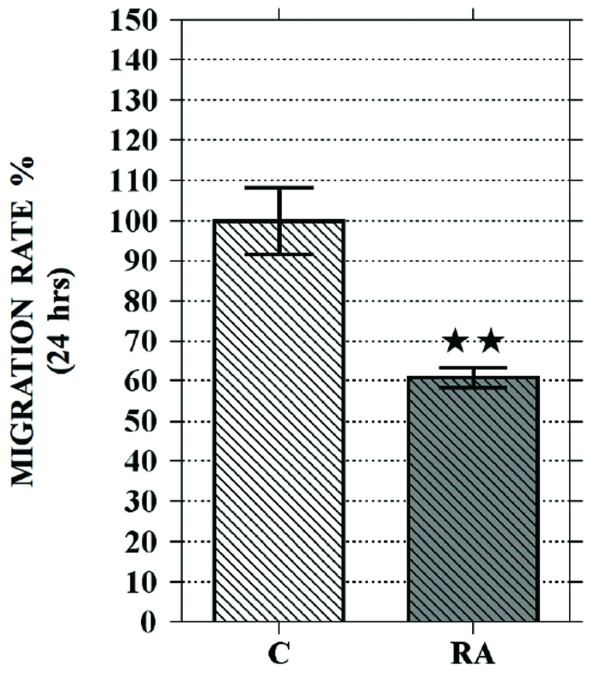
**Quantification of RA effect on neuroblastoma cell motility**. Graph shows that NB cell migration rate of 6 days RA treated cells (**RA**) is significantly reduced in comparison with untreated cells (**C**). Data are obtained from a set of scratch test analysis and are expressed as means ± standard error; N = 4;double star = p < 0.01.

Quantitative analysis clearly indicates a significant (about -40%) decrease of the cell migration rate following RA treatment (Fig. 7).

### Neuroblastoma cells with invasive activity are DCX^+^

**Figure 8:**
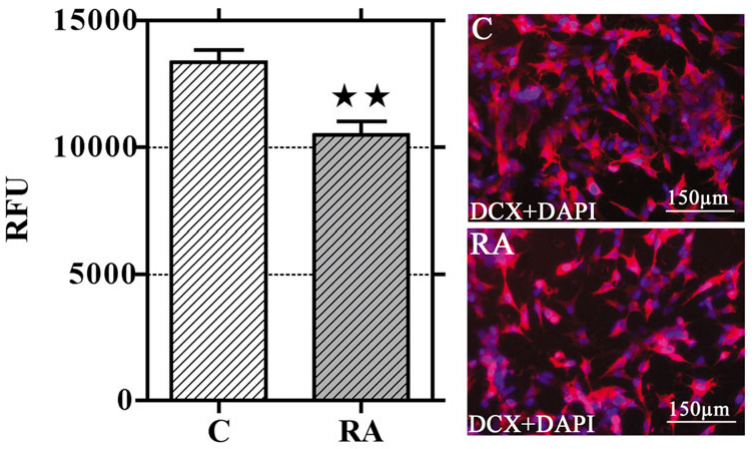
**RA treatment reduces SK-N-SH neuroblastoma cell invasiveness**. Invasiveness of NB cells is significantly reduced after 6 days RA treatment. The amount of invasive cells has been evaluated by means of a fluorescence assay (see Materials and Methods). Data are expressed as relative fluorescence units (RFU). Columns are means ± standard error; N = 4; double star = p < 0.01. Representative images of the cells on the bottom side of the insert filter show that all NB cells invading the matrix are DCX^+ ^(red) in both control and RA treated samples. Nuclei are counterstained with DAPI (blue).

NB cell invasiveness has been evaluated by means of a modified microchemotaxis assay. Cells cultured in normal conditions or treated with RA for 6 days were seeded into culture well containing polycarbonate membranes inserts coated with a film of Matrigel. After 48 hr, invasive cells migrated through the matrix-membrane unit and attached to the bottom of the membrane were dislodged and their number was estimated using a nuclear fluorescent dye. The results obtained show that RA treatment significantly reduces the amount of NB cell able to invade the Matrigel (chart of Fig. 8).

Some of the insert membranes have been processed for IF. Invading cells attached to the bottom of the membrane were fixed and immunostained for DCX. We found that, in both control and RA treated samples, all the cells migrated through the matrix/membrane unit are DCX^+^, even with different intensity (Fig. 8, right panels).

## Discussion

In the present study we demonstrate a differential expression of DCX in the cell subtypes composing human NB cell line SK-N-SH. DCX appears to be expressed exclusively in the cells that show high motility and invasiveness; these parameters are reduced by RA treatment that also down regulates DCX expression. It is noteworthy to say that after RA treatment all the cells that maintain motility and invasiveness express DCX.

DCX is a microtubule-associated protein (MAP) mainly involved in cell migration [10,12]. Modifications of DCX expression in neuron-like cell and its possible correlation with motility/invasion has been studied in mouse or in rat experimental models. For instance, it has been demonstrated that overexpression of DCX increases mouse neuronal migration rates *in vitro *[17]. On the contrary, targeted mutation or the shutdown of DCX disrupts the migration of neuronal cells [26,27].

Even though by the present data a direct involvement of DCX in these mechanisms cannot be assessed, to our knowledge this is the first evidence that in human NB cell line SK-N-SH DCX is specifically expressed in neuroblastic (N-type) cells, but it is absent in substrate adherent (S-type) cell type. Moreover, DCX is expressed in some, but not all, cells possessing an intermediate phenotype (I-type). Since it is sometime quite difficult to clearly distinguish the I-type from the N-type cells by their morphology, we found easier to identify the cells composing SK-N-SK cell line as DCX-expressing (DCX^+^) or DCX-not-expressing (DCX^-^) cells.

In addition, we show that also in NB cells this protein colocalizes with tubulin, as already reported for mouse and human migrating neurons [13,28,29].

The data here reported indicate for the first time that LIS1, another MAP that may dimerize with DCX in migrating neurons [16], is expressed in all the cell types composing SK-N-SH cell line. It is well known that DCX-LIS1 interaction plays a role in nucleus-centrosome coupling during neuronal migration [17]. Therefore, it is possible to postulate that LIS1-DCX interaction would play a role also in DCX^+ ^NB cell nucleokinesis and, possibly, in NB cell motility.

Moreover, here we report the presence of DCX in the two cell types (N- and I-type) that are indicated as the most malignant component of NB [2]. Actually, the results obtained by scratch-wound-healing assay and cell invasiveness test, clearly indicate that in control conditions all the cells that move into the scratched area and invade the Matrigel-coated membrane are DCX^+^. It is noteworthy to say that all the DCX^+ ^cells, the only that show motility and invasiveness, express LIS1; the solely expression of LIS1 in DCX^- ^cells excludes a role of it in spontaneous motility.

Of interest is the observation that VIM, an intermediate filament protein characteristic of non-neuronal and non-tumorigenic NB cell types [2,3], is present only in DCX^- ^and not in DCX^+ ^cells. This exclusive expression of DCX or VIM represents an additional parameter to identify different NB cell subtypes. Therefore, beside the well recognizable N-type and S-type cells, our results evidence at least three cell subtypes with a I-type morphology: DCX^+^, DCX^-^/VIM^+ ^and, in a small amount, cells lacking both DCX and VIM (DCX^-^/VIM^-^). There is not, at the moment, a clear explanation for the meaning of these three I-cell subtypes, however further studies are now in progress in the authors' lab.

RA treatment is known to cause differentiation of NB N-type cells into a more mature phenotype and to induce changes in the expression of specific markers [30,31].

Our data, while confirming previous evidences, show a progressive decrease of DCX expression during the RA-induced differentiation of N-type cells. As a consequence, the number of DCX^+ ^cells is greatly reduced by the treatment.

On the contrary, S-type cells are slightly affected by the treatment, and we did not observe any of these cells becoming DCX^+^. This observation confirms once more that RA acts specifically upon N-type cells [30,31]. In N-type cells, we observe that DCX level starts to decrease after 2 days of RA exposure and reaches the lowest value after 6 days of treatment. It should be noted that DCX level remains very low also after RA treatment withdrawal. Altogether, the results here reported indicate that DCX down-regulation starts very early after the onset of RA treatment, it is progressive, time-dependent, and irreversible.

Also LIS1 expression is reduced after RA treatment, but there are no cells completely lacking LIS1. Therefore, it can be argued that this protein is not directly correlated with cell differentiation.

In the present paper, we also report that SK-N-SH cell motility is greatly reduced by RA. The only cells able to move from the scratched borderlines into the wounded area are the few DCX^+ ^ones still present after the treatment. In accordance to scratch test results, we also demonstrate that RA exposure is able to reduce NB cell invasiveness; the only cells that invade the Matrigel-coated membrane are DCX^+^. This last observation underlines the importance of DCX as a marker for invasive NB cells.

The results here reported indicate that NB cell motility and invasiveness are characteristics of DCX^+ ^cells, and that, also after RA treatment, both of these capabilities are retained only by the cells expressing this protein.

Joshi and coworkers reported that 24 hr of RA exposure stimulates SH-SY5Y cell migration and invasiveness, while long term treatment (5 days) is less effective in stimulating migration but significantly reduces invasiveness [32]. Voight and co-workers [24] demonstrated that 3 days RA treatment does not modify cell migration of SK-N-FI, SH-SY5Y, SK-N-MC and SK-N-LO NB cell lines. In addition, SH-SY5Y cells showed a slight reduction in migratory capability whereas invasiveness was significantly reduced after 3 days of RA treatment [24]. In this regard, it is important to underline that our results on SK-N-SH motility have been obtained by scratching the cell monolayer after 6 days RA treatment, when DCX expression reaches the lowest values.

We have also observed that after RA treatment the cell motility is retained by the cells expressing both DCX and LIS1 but not by those cells expressing LIS1 alone.

Interestingly, we found that DCX and VIM signals may colocalize in the same N-type cell during short exposure to RA; however, after prolonged exposure, differentiated N-type cells loose DCX immunoreactivity and acquire a VIM positive phenotype. Although this effect is clearly evident by IF analysis, WB quantitation performed on the whole SK-N-SH cell population does not show VIM increase. This might probably be due to the concomitant slight decrease of VIM expression in S-type cells as observed by IF. Actually, using a NB cell line deprived of S-type cells, other authors have documented a significant increase of VIM expression after RA treatment [4]. Given the fact that motility and invasiveness are characteristics of DCX^+^/VIM^- ^cells, it is possible to postulate that VIM could be a marker not only of S-type cells [2,3], but also of those N-type cells that assume a DCX^-^/VIM^+ ^phenotype in response to RA treatment. This hypothesis is supported by the proposed role of VIM for axonal elongation in differentiating NB cells [33].

Finally, we have also observed that after RA treatment all the three I-cell subtypes (DCX^-^/VIM^-^; DCX^+^/VIM^-^; DCX^-^/VIM^+^) are still present. The presence of DCX^+ ^cells might indicate a resistance of these cells to RA treatment or the existence of a subpopulation of cells transiently expressing this protein during their differentiation process. Actually, it has been demonstrated that during adult hippocampal neurogenesis DCX is not expressed by stem cells, but only by neural progenitor cells and disappears when progenitor cells differentiate to mature neurons [34]. We may therefore suppose that RA could induce the differentiation of DCX^-^/VIM^- ^I-type cells toward a DCX^-^/VIM^+ ^phenotype through an intermediate phase characterized by DCX expression. Further studies will be addressed to specifically test this hypothesis.

Besides the observation that DCX is a marker for the minimal residual disease in human neuroblastoma [6], our results provides the new evidence that this protein is not present in all cell subtypes composing SK-N-SH cell line but it is express only in the more aggressive neuroblastoma cell subpopulation and in the retinoic acid-resistant cells that possess high motility and invasiveness.

## Conclusion

We report the first demonstration that DCX is differentially expressed only in some (N and I) cell types composing SK-N-SH human NB cell-line.

All the DCX^+ ^cells show high migratory capability and invasiveness; moreover, we show that RA treatment induces a significant reduction of these parameters and down-regulates DCX expression.

RA is unable to completely block DCX expression in all the cells, and the few DCX^+ ^cells that are still present after the treatment show high motility and invasiveness.

We found that the some DCX^+ ^RA-resistant cells have the morphological features of I-type cells, and since I-type cells are indicated as the more undifferentiated and aggressive cellular component of human NB, the presence of these cells after the treatment might support the emerging concept about the existence of cancer stem cells liable to tumor relapse [35]. This last hypothesis is presently under investigation.

Irrespective of a direct role of DCX in NB cell motility, the present data suggest that the detection of DCX^+ ^cells in untreated and treated tumors in neuroblastoma affected patients may be prognostic of tumor aggressiveness, as well as of a severe tumor relapse after treatment.

## Abbreviations

NB: Neuroblastoma; DCX: Doublecortin; LIS1: Lissencephaly-1; VIM: Vimentin; NF-68: Neurofilaments-68; IF: Immunofluorescence; PC: Phase contrast; CIP: Calf intestine alkaline phosphatase; WB: Western blot; RA: Retinoic Acid.

## Competing interests

The author(s) declare that they have no competing interests.

## Authors' contributions

EM and MCF carrier out all the experiments. CC performed sequence analysis of the DCX gene. MZ and RM were responsible for the progress of the work. All contributors participated in the conception and design of the study and have been involved in the writing of the manuscript.

## Pre-publication history

The pre-publication history for this paper can be accessed here:


